# Targeting the Epigenetic Landscape for Lung Cancer Treatment

**DOI:** 10.1111/jcmm.70425

**Published:** 2025-02-10

**Authors:** Kostas A. Papavassiliou, Amalia A. Sofianidi, Antonios N. Gargalionis, Athanasios G. Papavassiliou

**Affiliations:** ^1^ First University Department of Respiratory Medicine, ‘Sotiria’ Chest Hospital, Medical School National and Kapodistrian University of Athens Athens Greece; ^2^ Department of Biological Chemistry, Medical School National and Kapodistrian University of Athens Athens Greece; ^3^ Laboratory of Clinical Biochemistry, ‘Attikon’ University General Hospital, Medical School National and Kapodistrian University of Athens Athens Greece

**Keywords:** DNA methylation, epigenetic targeting, epigenome, histone modification lung cancer

The term ‘epigenetics’ was introduced in 1942 by embryologist Conrad Waddington [1]. However, it was not until the early 21st century that the concept began to attract widespread attention. The field reached a pivotal moment in 2022 when Douglas Hanahan identified epigenetic alterations as a fundamental hallmark of cancer, emphasising their critical contribution to tumour development and progression [2]. Notably, lung cancer is characterised by both intra‐ and intertumoral heterogeneity, driven by genetic changes alongside epigenetic modifications [3]. Due to the extensive and multifaceted role of epigenetic regulation in lung cancer, targeting the reversible nature of the epigenome presents a promising therapeutic approach to address the complexity of tumour heterogeneity, which has long posed serious challenges in lung cancer treatment. Herein, we highlight recent advances in epigenome‐targeting strategies in the highly demanding field of lung cancer therapeutics.

The antitumor potential of histone deacetylase (HDAC) inhibitors has been well recognised for almost 20 years [4]. Suberoylanilide hydroxamic acid (SAHA; commonly known as vorinostat) is a leading pan‐HDAC inhibitor of class I/II HDAC enzymes (HDAC1/2) with a demonstrated favourable safety profile in a phase I clinical trial involving patients with *Bcl‐2‐like protein 11* (*BIM*) deletion‐containing non‐small cell lung cancer (NSCLC) resistant to epidermal growth factor receptor (EGFR) tyrosine kinase inhibitor (TKI) therapy [5]. Hydroxamic acid‐derived HDAC inhibitors, such as trichostatin A (TSA) and quinostat, have shown promising preclinical efficacy against NSCLC [6]. These compounds induce alterations in tight junction proteins of human lung adenocarcinoma cells while preserving the integrity of the normal epithelial barrier in healthy cells [6].

Other compelling targets within the epigenome of lung cancer are DNA methyltransferases (DNMTs). In a phase I study, the DNMT inhibitor azacitidine (5‐azacytidine) demonstrated a reduction in global DNA methylation of the bronchial epithelium following aerosolised treatment in NSCLC patients [7]. The treatment was associated with negligible plasma concentrations of the DNMT inhibitor, which indicate minimal systemic absorption of the drug, and thus, exhibited excellent tolerability [7]. Another DNMT inhibitor, decitabine (a deoxycycline and cytarabine nucleotide derivative), was recently found to preclinically abate NSCLC cell growth and metastatic potential when co‐administered with aspirin by inhibiting the β‐catenin/signal transducer and activator of transcription 3 (STAT3) signalling axis [8]. An additional drug influenced by DNMTs is temozolomide, an oral alkylating agent that interferes with DNA through the ability of its metabolites to deposit methyl groups on DNA guanine bases and is removed from the system by a specific DNMT [9]. A phase I/II trial of temozolomide combined with the poly‐ADP ribose polymerase (PARP) inhibitor olaparib reported that this combination shows promise as an effective treatment modality for relapsed small cell lung cancer (SCLC), particularly in patients with brain metastases [10]. Remarkably, PARP inhibitors have lately been combined with protein arginine methyltransferase (PRMT) inhibitors. PRMTs are epigenetic regulators, frequently overexpressed in various types of cancers, including NSCLC. The synergistic effects of type I PRMT and PARP inhibitors against NSCLC cells highlight a promising novel route in lung cancer therapeutics [11]. Tightly associated with the process of methylation, lysine‐specific histone demethylase 1A (LSD1; a flavin‐dependent monoamine oxidase) functions as an epigenetic eraser, removing methyl groups from histone lysines and fostering tumorigenesis. A recent study unveiled pyrrolo[2,3‐*c*]pyridines as a new class of extremely potent and reversible LSD1 inhibitors, which managed to hamper the growth of a SCLC cell line in vitro [12].

Targeting the epigenetic processes in lung cancer has also proven to be effective at enhancing antitumor immunity. Combining inhibition of the enzymatic subunit enhancer of zeste homologue 2 (EZH2), which is involved in the methylation of histones and agonism of the stimulator of interferon response cGAMP interactor (STING) has been shown to trigger an immune response against SCLC cells [13]. A recent study revealed that the disialoganglioside GD2, a glycosphingolipid subtype, is abnormally expressed both in SCLC and NSCLC and can be targetable through chimeric antigen receptor (CAR) T‐cell therapy. Tazemetostat, a novel EZH2 inhibitor, could be used to up‐regulate GD2 expression in lung tumour cells, amplifying their responsiveness to CAR T‐cell targeting [14]. Similarly, targeting bromodomain and extra‐terminal (BET) family proteins, which are vital for chromatin remodelling and are implicated in permissive physical interactions with epigenetically modified histones, coregulators and oncogene control elements [15], alongside T‐cell bispecific antibodies or immune checkpoint blockade, facilitates antitumor responses through a tumour necrosis factor (TNF)‐dependent mechanism [16]. An orally bioavailable, second‐generation, potent pan‐BET inhibitor, ZEN‐3694, is under clinical investigation in a phase II trial (NCT05607108) in patients with advanced squamous cell carcinoma (SCC) harbouring multiple copies (amplification) of the *nuclear receptor‐binding SET domain protein 3* (*NSD3*) gene, which encodes a key SET domain‐containing histone lysine methyltransferase. Furthermore, the HDAC1/2 inhibitor FK228 displayed enhanced tumour cell sensitivity to natural killer (NK) cell‐mediated cytotoxicity [17]. Lastly, the combination of the HDAC inhibitor TSA with an immune checkpoint inhibitor has demonstrated high effectiveness, significantly enhancing the persistence of antitumor immune responses in vitro [18].

Lately, various natural substances and their derivatives have been explored as epigenetic modifiers regarding their efficacy to mitigate lung cancer growth and development. Growing evidence suggests that curcumin, a phenolic compound produced by plants of the *Curcuma longa* species, may exhibit epigenetic regulatory effects on microRNAs (miRNAs). According to a recent study, curcumin up‐regulates miR‐192‐5p while suppressing the expression of DNA methylation‐engaged enzymes, including DNMT1, DNMT3A and DNMT3B, in NSCLC cells. In this context, curcumin functions as a chemosensitiser, increasing the cytotoxicity of the TKI crizotinib and inducing apoptosis in lung tumour cells [19]. Another noteworthy natural agent is cucurbitacin B, a triterpenoid derived from the Cucurbitaceae family of flowering plants, known for its diverse bioactivities. It was recently reported that cucurbitacin B augments the expression of miR‐let‐7c through the suppression of the interleukin‐6 (IL‐6)/STAT3 pathway. Notably, inhibition of the X inactive specific transcript (XIST)/miR‐let‐7c/IL‐6/STAT3 axis promoted the apoptosis of lung cancer cells and suppressed their proliferation [20], opening new avenues for the application of regimens based on natural compounds in lung cancer therapeutics.

Following the affixation of the tumour microenvironment (TME) to the intricate setting of cancer, it has become clear that tumour cells actively interact with its various components to drive tumour progression. A promising new strategy for halting lung cancer development involves targeting the epigenetic landscape of TME components, particularly cancer‐associated fibroblasts (CAFs). Various HDAC inhibitors have proven effective at halting the tumorigenic capacity of CAFs. Dual inhibition of phosphoinositide 3‐kinase (PI3K) class I isoforms and HDAC by the orally available small molecule fimepinostat, thereby preventing the activation of the PI3K‐AKT‐mechanistic target of rapamycin (mTOR) signal transduction pathway, has yielded positive results in vivo by restraining the migration and invasion properties of CAFs in NSCLC models [21]. A recent study explored an indirect approach to target CAFs by focusing on paracrine molecules involved in their activation and epigenetic modulation. LY2109761, a potent and orally active inhibitor of transforming growth factor beta (TGF‐β) receptor type 1 (TGFBR1), demonstrated effectiveness in reducing the expansion of SCC‐associated CAFs in vivo [22]. Targeting key transcription factors of lung CAFs, such as the hypermethylated SMAD3 [23], or non‐coding RNAs like miRNAs, presents significant potential in curbing the evolution of lung tumours.

Evidently, employing epigenetic treatment modalities in lung cancer holds substantial promise. The epigenome of lung cancer remains only partially understood, highlighting the need for further research to unlock its full therapeutic potential. Nevertheless, one of the main challenges in developing epigenome‐targeted therapies lies in the considerable variability of the epigenome, both between individuals and within the same individual over time and in response to different treatments. To design effective, rationally guided strategies, it is essential to map the epigenome of each patient individually. While this process can be costly, the use of artificial intelligence (AI) could offer a time‐efficient solution to unravel the unique epigenetic profile of each patient, potentially revolutionising curative personalised treatment options.

## Author Contributions

**Kostas A. Papavassiliou:** conceptualization (lead), data curation (lead), writing – original draft (lead). **Amalia A. Sofianidi:** conceptualization (equal), data curation (equal), writing – original draft (equal). **Antonios N. Gargalionis:** conceptualization (equal), data curation (equal), writing – original draft (equal). **Athanasios G. Papavassiliou:** conceptualization (lead), data curation (lead), supervision (lead), writing – review and editing (lead).

## Conflicts of Interest

The authors declare no conflicts of interest.

## Data Availability

Data sharing not applicable‐no new data generated.
